# School-based screening for scoliosis and foot biomechanical abnormalities: a study of prevalence, plantar pressure, and gait among adolescents in Haidian District, Beijing

**DOI:** 10.3389/fped.2026.1749070

**Published:** 2026-04-01

**Authors:** Zelong Miao, Yuanyuan Xue, Chengxin Li, Chaohui Xing, Hao Jiang, Tianxiang Wang, Dongyuan Zhang, Qian Li, Yi Liu, Chenxi Wang, Yuansong Zhao, Zhong Zhang, Dechen Song, Bin Jia

**Affiliations:** 1 Biology, School of Teacher’s Education, Hainan Normal University, Haikou, China; 2 Beijing DCN Orthopaedic Hospital, Beijing, China; 3 MOE Key Laboratory of Bioinformatics, Center for Synthetic and Systematic Biology, School of Life Sciences, Tsinghua University, Beijing, China; 4 Tsinghua-Peking Center for Life Sciences, Beijing, China; 5 Department of Nutrition and Health, China Agricultural University, Beijing, China

**Keywords:** adolescents, biomechanics, gait analysis, plantar pressure, scoliosis

## Abstract

**Background:**

Adolescent idiopathic scoliosis (AIS) and foot biomechanical disorders are significant public health concerns, impacting physical function and quality of life. Despite national screening mandates, regional data remain sparse.

**Methods:**

We conducted a school-based screening study to investigate the prevalence of scoliosis and foot-related biomechanical abnormalities among adolescents in Haidian District, Beijing, China. A total of 1,419 participants (739 males and 680 female) from primary and middle schools underwent spinal and plantar health assessments using standardized protocols, including the Adams forward bend test with a scoliometer, Foot Posture Index-6 (FPI-6), and static/dynamic plantar pressure analysis.

**Results:**

This cross-sectional study of 1,419 Beijing adolescents revealed a 2.04% prevalence of scoliosis (29 cases), showing significant female predominance (3.53% vs. 0.68%; *χ*^2^ = 14.397, *p* < 0.001, *φ* = −0.101, Cramer's V = 0.101). Scoliosis prevalence showed distinct developmental patterns, with a significant increasing trend across grades (*p* = 0.011). Clear peaks were observed in 6th (4.22%) and 9th (4.35%) grades, where cases significantly exceeded expected counts, though the reliability of estimates for upper grades (11th-12th) may be limited by smaller sample sizes (48 and 18 participants, respectively). In contrast, the detection rate of foot abnormalities was slightly higher among males (47.8% vs. 43.4%), this difference did not reach statistical significance (*p* = 0.098), and the effect size was negligible (Phi = 0.044). This indicates that foot abnormalities represent a common health concern affecting both sexes. Significant variation in foot abnormality prevalence was observed across grades [*χ*^2^(11) = 36.98, *p* < 0.001], peaking sharply at school entry (62.7% in 1st grade) and then declining rapidly through elementary school. Prevalence stabilized during middle adolescence, with a non-significant apparent rebound in the small 12th-grade sample requiring cautious interpretation.

**Conclusions:**

Key findings highlight the need for early and targeted interventions within school health programs. A gender-stratified approach is recommended: implementing gender-sensitive screening for scoliosis, while adopting universal promotion measures for foot health. Specific priorities include increasing flatfoot screening frequency among 1st–2nd grade boys and enhancing posture-related health education for adolescent girls during key transitional growth phases. These strategies aim to mitigate long-term musculoskeletal complications.

## 1 Introduction

Adolescent Idiopathic Scoliosis (AIS) is a complex three-dimensional spinal deformity characterized by lateral curvature in the coronal plane (Cobb angle >10°), sagittal imbalance, and varying degrees of axial rotation, typically manifesting during adolescence (1–4). Globally, the prevalence ranges from 0.47% to 5.20% (5), with females affected 2–10 times more frequently than males (6). In China, a large-scale screening of 1,149,330 adolescents aged 10–18 years revealed an overall prevalence of 1.2%, with a rising trend observed in recent years. This increase may be attributed to the growing implementation of spinal screening programs, lifestyle factors such as prolonged sitting and increased electronic device usage among adolescents, as well as earlier growth spurts in females—who already exhibit a higher prevalence (1.6%) compared to males (1.0%). Additionally, older adolescents (16–18 years) showed a slightly higher prevalence (1.3%) than their younger counterparts (10–15 years, 1.1%), which may reflect the progressive nature of the condition and cumulative postural influences during growth (7).

Clinically, AIS often presents with severe back pain, restricted range of motion, and trunk asymmetry. Without intervention, the deformity may progress, leading to functional disability and severe complications such as cardiovascular impairment, reduced pulmonary function, chronic pain, and psychological distress (8, 9). Additionally, moderate-to-severe AIS imposes psychological burdens on patients and significant socioeconomic costs on families and healthcare systems (10). The etiology of AIS remains multifactorial (11), involving genetic predisposition (e.g., SPRY4 gene mutations, which impair osteogenic differentiation and melatonin response in bone marrow-derived mesenchymal stem cells, linking genetic and hormonal dysregulation to spinal deformity (12, 13), endocrine abnormalities affecting bone metabolism (14–16), biomechanical factors (17, 18), neuromuscular mechanisms (19), and aberrant mesenchymal stem cell differentiation (20–25). AIS exhibits a polygenic inheritance pattern, as evidenced by the Carter effect, which describes the increased severity and familial aggregation observed primarily through female relatives. Beyond this pattern, specific genetic loci have been implicated through genome-wide association studies (GWAS) and candidate gene analyses. In addition to the SPRY4 gene, other susceptibility genes such as LBX1 and GPR126 have been consistently associated with AIS. Furthermore, genes like WFS1, which is critical for mitochondrial function and cellular growth regulation, have also been linked to spinal development anomalies (66). Emerging evidence also points to the role of epigenetic mechanisms, including the influence of repetitive genetic elements on bone metabolism and spinal stability (26), highlighting another layer of regulatory complexity in spinal development. Collectively, these genetic and epigenetic factors establish a biological predisposition, upon which environmental triggers—such as biomechanical stress, hormonal changes during puberty, and lifestyle factors—may act to initiate or exacerbate the spinal curvature.

Due to its complex pathogenesis and rapid progression, early detection and intervention are critical. Current screening protocols recommend using a scoliometer to measure the angle of trunk rotation (ATR) (27–29). If ATR exceeds 5°, radiographic confirmation (Cobb angle >10°) is advised. Studies demonstrate that scoliometer measurements exhibit high sensitivity (>92%) for detecting thoracic curves >20°, with a strong positive correlation between ATR and Cobb angle (29).

Research indicates that AIS is associated with altered plantar pressure distribution and abnormal gait patterns (30). These foot biomechanical impairments may contribute to postural imbalance and potentially influence the progression of AIS (31). Consequently, foot health represents a critical yet understudied dimension of musculoskeletal development in adolescents. As the foundation of posture, locomotion, and physical activity, foot structure and function significantly impact autonomy, independence, and overall well-being (32, 33). Foot disorders have emerged as a major public health challenge, with reported prevalence rates ranging from 61% to 79% and continuing to rise (34, 35). Common conditions such as foot pain, deformities, muscle weakness, and restricted motion can substantially diminish quality of life (36–38).

Among these, flatfoot is one of the most frequently encountered conditions in pediatric foot clinics. It is characterized by collapse or excessive flattening of the medial longitudinal arch (39) and is primarily attributed to abnormal bone structure or ligamentous laxity leading to arch insufficiency (40). While most cases respond well to conservative management, only a small proportion require surgical correction (41). Beyond anatomical factors, external influences such as physical activity levels, footwear habits, and environmental factors also contribute significantly to the development of flatfoot in children and adolescents (42). The pediatric flatfoot often raises parental concern due to the potential for symptoms such as fatigue and pain, or progression to pathological forms (43). Persistent arch abnormalities accompanied by pain or skeletal deformities can markedly reduce health-related quality of life. Studies consistently report a higher prevalence of flatfoot in males, with rates of approximately 52% in males compared to 36% in females (44). In addition to gender, factors such as age (45–47), geographic region (48–50), lifestyle and dietary habits (51), obesity (44, 46, 52–54), and physical activity levels (46) are associated with flatfoot occurrence. In contrast, pes cavus (high-arched foot) is more common among females, with higher prevalence observed in children aged 4–13 years (55).

Plantar pressure assessment and gait analysis serve as essential tools for detecting biomechanical abnormalities that may predispose individuals to long-term musculoskeletal complications, including flatfoot, scoliosis, and osteoarthritis (56). These methods also allow objective evaluation of conditions such as varus/valgus deformities, pronation/supination imbalances, and in-toeing/out-toeing gait patterns. Foot disorders not only diminish quality of life and impede daily activities but also elevate the risk of falls and related injuries (57–61).

Despite global recognition of these issues, comprehensive epidemiological data on scoliosis prevalence, plantar pressure characteristics, and gait abnormalities among adolescents in Beijing remain limited. Based on a district-wide screening of 1,419 adolescents in Beijing using standardized scoliometer, plantar pressure assessment, and gait analysis, this study reveals a low prevalence of scoliosis (2.04%) with significant female predominance, contrasting sharply with a high prevalence of foot biomechanical abnormalities (45.67%) which predominantly affect males and younger students. These distinct gender- and grade-specific patterns provide crucial regional epidemiological data, highlight the necessity of implementing targeted school screening programs, and establish a foundational methodology and baseline for future longitudinal research on the interplay between spinal and pedal health in adolescent populations.

## 2 Methods

### 2.1 Study participants and design

A school-based cross-sectional survey was conducted in Haidian District, Beijing. A cluster sampling method was employed, in which several primary and secondary schools were randomly selected from the list of schools in the district. Within selected school, students from grades 1 through 12 were invited to participate. Prior to the screening, detailed information about the study was distributed to parents or legal guardians through school communications. Written informed consent was obtained from all participants and their guardians after a thorough explanation of the study procedures. All assessments were conducted in accordance with the ethical guidelines approved by the Ethics Committee of Beijing DCN Orthopedic Hospital (Approval No. JD2022-02).

A total of 1,419 students (739 males, 680 females) aged 6–18 years were included in the final analysis. The distribution of participants across grade levels was as follows: 142 first graders, 126 s graders, 139 third graders, 149 fourth graders, 181 fifth graders, 166 sixth graders, 128 seventh graders, 111 eighth graders, 115 ninth graders, 96 tenth graders, 48 eleventh graders, and 18 twelfth graders (Supplementary Table S1). The male-to-female ratio (1.09:1) was balanced and comparable to the overall gender distribution reported in the Haidian District education statistics.

While this sampling strategy provided comprehensive coverage of key developmental stages from childhood through late adolescence, the relatively small sample sizes in the 11th (*n* = 48) and 12th (*n* = 18) grades may reduce the reliability and precision of prevalence estimates for these specific cohorts. Therefore, findings pertaining to these upper grades should be interpreted with caution.

### 2.2 Screening methods for scoliosis

The screening process for scoliosis involved a combination of visual inspection and quantitative assessment using a scoliometer. Initial evaluation was performed through visual observation to assess shoulder and pelvic symmetry, as well as spinal alignment. Participants were examined in a standing position to detect any visible asymmetry in shoulder height, iliac crest level, or spinal curvature.

For more precise measurement, the Adams forward bend test was conducted using a scoliometer to determine the degree of trunk rotation, which correlates with spinal curvature. Participants stood with their feet together and palms opposed, then flexed their shoulders to 90 degrees while bending forward at the spine. The scoliometer was placed along the spine to measure rotational asymmetry at each vertebral level. Scoliometer readings were interpreted based on established correlations with radiographic Cobb angles. A measurement of ≤5° indicated a spinal curvature of <20°(Cobb angle) with 95% sensitivity, corresponding to an average Cobb angle of 11°. A reading of ≤7° suggested a curvature of <25° (average 20°) with 88% sensitivity. The device demonstrated high reliability, as 95% of curves exceeding 20° (Cobb angle) were detected at a scoliometer threshold of ≥7°. This approach offered a rapid and easy-to-operate method for scoliosis screening in clinical and school-based settings, making it suitable for initial screening without the need for x-ray examination.

### 2.3 Screening methods for plantar health

This study utilized the FreeMed™ plantar pressure and posture assessment platform (Sensor Medica s.a.s., Italy), a 1.2 m × 0.8 m pressure distribution system with high-sensitivity sensors and a sampling frequency >400 Hz. For static assessment, participants stood naturally on the platform for 3–4 s. For dynamic gait analysis, participants walked naturally along a walkway for five round trips (62). The screening protocol was designed based on professional biomechanical theory and the target population's age range, utilizing the Foot Posture Index-6 (FPI-6) as a foundational framework. The FPI-6 is a clinically validated tool for quantifying static foot alignment and postural abnormalities, with established reliability, validity, sensitivity, and specificity. Participants were assessed in the Relaxed Calcaneal Stance Position (RCSP). Three key observational parameters recorded: (1) the frontal-plane angle of the calcaneus relative to the tibia, (2) the visibility of lateral toes from a posterior view (indicative of subtalar pronation), and (3) forefoot morphology, including the presence of Morton's Foot (characterized by a hypoplastic first metatarsal and elongated second/third metatarsals) or transverse arch collapse.

The combined findings of (1) and (2) were used to classify subtalar joint alignment as excessive pronation, excessive supination, or neutral (no significant deviation). This classification was further interpreted through a structured pain history interview, documenting the location, nature, duration, and triggers of lower-limb movement-related pain, along with aggravating or alleviating factors. Forefoot structural anomalies (e.g., Morton's Foot or transverse arch collapse) were evaluated for their association with active or historical pain to identify potential injury mechanisms.

Participants were categorized as normal if they exhibited neutral subtalar alignment without movement-related pain. Those with subtalar deviation (pronation/supination), forefoot anomalies, or both were stratified by pain status: asymptomatic individuals were advised to monitor for symptoms, while those with active pain were referred for advanced biomechanical assessment (e.g., plantar pressure or gait analysis). Individuals with prior pain history but no current symptoms underwent gait lab evaluation to determine the need for custom orthotic intervention.

### 2.4 Data integration and statistical analysis

Statistical analyses were performed using SPSS Statistics version 27.0 and graphing was conducted using GraphPad Prism software, version 9.0. Descriptive statistics were calculated for demographic and clinical variables, with prevalence rates presented as percentages. For comparisons of prevalence between two independent groups (e.g., male vs. female), the Chi-square test or Fisher's exact test was applied, as appropriate. Comparisons of prevalence across multiple independent groups (e.g., different grade levels) were conducted using the Chi-square test for trend. All key prevalence estimates were reported with their corresponding 95% confidence intervals (CIs). Where statistically significant differences were identified, effect sizes were calculated and reported to evaluate the magnitude of the observed associations: Phi coefficient or Cramer's V for categorical data. The threshold for statistical significance was set at *α* = 0.05 for all tests.

## 3 Results

### 3.1 Scoliosis prevalence

The comprehensive screening of 1,419 adolescents (739 males and 680 females) in Beijing revealed a 2.04% overall scoliosis prevalence (29 cases), demonstrating significant gender disparities (males: 0.68% [5/739]; females: 3.53% [24/680]) (Table 1) (*χ*^2^ = 14.397, *p* < 0.001, *φ* = −0.101, Cramer's V = 0.101). Prevalence followed distinct developmental patterns, with the lowest rates in elementary grades (0.67–0.79% in grades 1–4) and peak occurrences during pubertal transition periods: 4.22% in 6th grade, 4.35% in 9th grade, and 5.56% in 12th grade. The Chi-Square test suggested variation in prevalence across grades (Pearson *χ*^2^ = 17.323, *p* = 0.099), though it did not reach the conventional level of statistical significance (*p* < 0.05). However, a significant linear-by-linear association was found (*χ*^2^ = 6.503, *p* = 0.011), indicating a statistically significant increasing trend in scoliosis prevalence with advancing school grade. The standardized residual for 6th grade was 2.0, indicating that the observed number of cases (*n* = 7) was significantly higher than the expected count (3.4) based on the overall prevalence (|residual| > 1.96). The standardized residual for 9th grade was 1.7, approaching the significance threshold, still suggesting a higher-than-average prevalence (4.35%, *n* = 5). While not statistically significant, the prevalence in 10th grade (residual 1.5, 4.17%) and 5th grade (residual 0.7, 2.76%) were numerically higher than in the lower grades (1st-4th, 0.67%–0.79%). These findings indicate critical periods of postural vulnerability during pubertal development. The significant rise in prevalence beginning in grades 5–6 appears to coincide with both the typical onset of adolescent growth spurts in this population and concurrent lifestyle changes, including increased academic demands leading to prolonged sedentary study habits and reduced physical activity (63, 64).

**Table 1:** Prevalence of scoliosis and foot abnormalities by school and gender among Beijing adolescents.

Abnormalities categorized by grade	Total number	Number of people with scoliosis	Male	Female	Scoliosis ratio (%)	Number of people with foot abnormalities	Male	Female	Foot abnormalities ratio (%)
1st (Elementary School)	142	1	0	1	0.70	89	56	33	62.68
2nd (Elementary School)	126	1	0	1	0.79	64	40	24	50.79
3rd (Elementary School)	139	1	1	0	0.72	60	30	30	43.17
4th (Elementary School)	149	1	0	1	0.67	68	25	43	45.64
5th (Elementary School)	181	5	2	3	2.76	96	56	40	53.04
6th (Elementary School)	166	7	2	5	4.22	66	29	37	39.76
7th (Junior High School)	128	1	0	1	0.78	51	26	25	39.84
8th (Junior HighSchool)	111	1	0	1	0.90	48	32	16	43.24
9th (Junior HighSchool)	115	5	0	5	4.35	43	25	18	37.39
10th (Senior High School)	96	4	0	4	4.17	36	18	18	37.50
11th (Senior High School)	48	1	0	1	2.08	16	12	4	33.33
12th (Senior High School)	18	1	0	1	5.56	11	4	7	61.11
Total number	1,419	29	5	24	2.04	648	353	295	45.67

Frequency distribution of scoliosis and foot abnormalities among 1,419 Beijing adolescents (grades 1–12) stratified by gender and school level (elementary/junior high/senior high).

**Figure 1:**
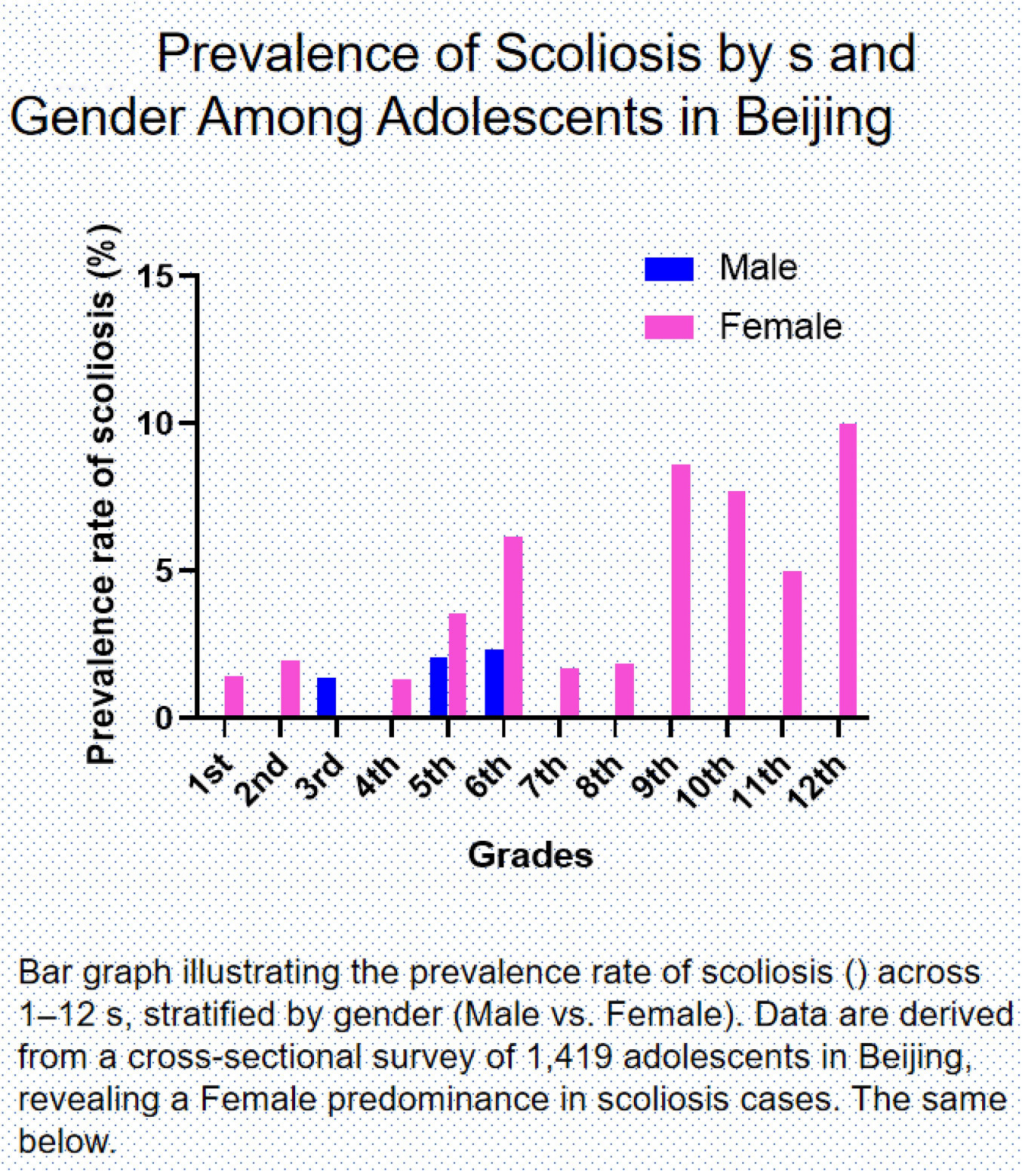
Prevalence of scoliosis by s and gender Among adolescents in Beijing. Bar graph illustrating the prevalence rate of scoliosis () accross 1–12 s, stratified by gender (Male vs. Female). Data are derived from a cross-sectional survey of 1,419 adolescents in Beijing, revealing a Female predominance in scoliosis cases. The same below.

Furthermore, gender differences became increasingly pronounced with advancing school levels—while male cases were confined to grades 3, 5-6 (maximal rate: 2.35% in 6th grade), female prevalence escalated dramatically in secondary education, reaching 8.62% in 9th grade, and 7.69% in 10th grade (Figure 1). An additional 11 borderline cases (3 males, 8 females) were identified as requiring further diagnostic follow-up (Table 1). Overall, the analysis revealed significant differences in scoliosis prevalence by gender and grade, with females consistently showing higher rates across most grades. These findings highlight the importance of implementing targeted screening strategies during key developmental periods.

**Figure 2:**
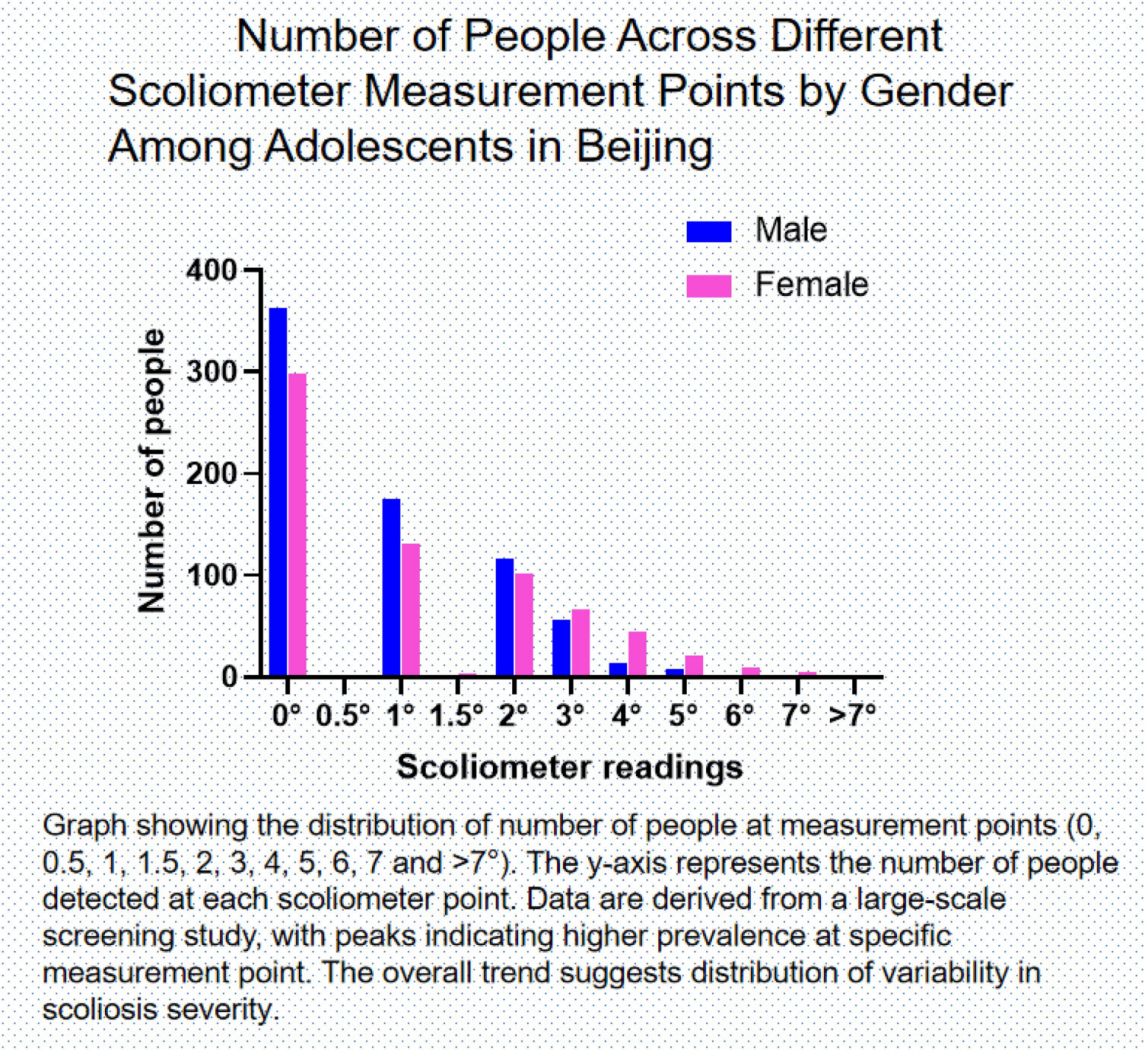
Number of people across different scoliometer measurement points by gender Among adolescents in Beijing. Graph showing the distribution of number of people at measurement points (0, 0.5, 1, 1.5, 2, 3, 4, 5, 6, 7 and >7°). The *y*-axis represents the number of people detected at each scoliometer point. Data are derived from a large-scale screening study, with peaks indicating higher prevalence at specific measurement point. The overall trend suggests distribution of variability in scoliosis severity.

The distribution of scoliometer measurements among Beijing adolescents revealed distinct patterns in spinal alignment characteristics, with the majority of participants (46.65%, *n* = 662) demonstrating perfect spinal alignment (0°). Minor spinal deviations were common, with 306 cases at 1° and 218 cases at 2°, collectively representing 37.14% (1°-2°) of the screened population (Figure 2). The prevalence of clinically significant spinal curvatures (≥3°) progressively declined, with 122 cases at 3° (8.60%), 59 at 4° (4.16%), 29 at 5° (2.04%), 9 at 6° (0.63%), 7 at 7° (0.49%), and only 3 cases exceeding 7° (0.21%) (Table 2 and Supplementary Table S2).

**Table 2:** Distribution of scoliometer readings by gender and s among adolescents in Beijing.

Scoliometer readings (°)	Total number	M	F	1st	2nd	3rd	4th	5th	6th	7th	8th	9th	10th	11th	12th
0°	662	363	299	58	59	81	66	92	92	50	55	40	34	22	13
0.5°	1	1	0	0	1	0	0	0	0	0	0	0	0	0	0
1°	306	175	131	34	27	26	34	34	30	42	22	23	21	10	3
1.5°	3	0	3	1	1	1	0	0	0	0	0	0	0	0	0
2°	218	116	102	22	23	23	23	23	18	18	19	21	17	10	1
3°	122	56	66	18	9	3	12	10	13	11	10	18	15	3	0
4°	59	14	45	5	2	3	7	13	6	6	4	7	5	1	0
5°	29	8	21	3	2	1	4	5	4	0	0	5	3	1	1
6°	9	0	9	0	1	1	2	1	1	1	0	0	1	1	0
7°	7	2	5	1	1	0	1	3	0	0	0	1	0	0	0
>7°	3	1	2	0	0	0	0	0	2	0	1	0	0	0	0

Frequency table displaying the number of participants across different scoliometer measurement angles (°), stratified by gender (M/F) and school (1st–12th). Data are derived from a cross-sectional screening of Beijing adolescents (Total *N* = 1,419). M: Male, F: Female. The same below.

Gender-specific analysis demonstrated a female predominance in higher-angle measurements, with females accounting for 73.83% of cases with curvatures ≥4°, compared to 26.17% in males. Notably, all 6° cases (*n* = 9) occurred in females, whereas males showed a relative predominance at 7° (71.43%). Grade-level distribution analysis revealed two peaks in ≥4° curvature prevalence: one among elementary school students (grades 4–6, accounting for 13.08%, 20.56%, and 12.15%, respectively) and another in 9th graders (12.15%). The lower detection rate in 11th and 12th grades (2.80% combined) may reflect smaller sample sizes in these cohorts (Table 2, Supplementary Table S3).

Subgroup analysis identified specific patterns: 5th-grade males were most prevalent among 4° cases (18.64%), 9th-grade females were predominant in 5° cases (17.24%), 4th-grade females accounted for 22.22% of 6° cases, and 5th-grade males represented 28.57% of 7° cases. Among the most severe cases (>7°), 6th-grade students (both sexes) and 8th-grade females each accounted for 33.33% (Supplementary Table S4), suggesting these subgroups may require particular attention for spinal health.

The data revealed three key epidemiological trends: a progressive female predominance with increasing curvature severity (gender ratio shifting from 1:1 at 1° to 9:0 at 6°), a bimodal clustering of severe curvatures (>7°) in elementary and middle school grades, and a predominant maintenance of spinal symmetry, with 92.46% of participants showing ≤3° measurements (Table 2). These findings establish quantitative evidence for spinal alignment assessment in Chinese adolescents and highlight critical periods for monitoring spinal development during pubertal growth.

### 3.2 Foot abnormalities

The study revealed a high prevalence of foot abnormalities among 1,419 adolescents in Beijing, with 45.67% (648 cases) affected. Overall, males showed a higher detection rate (47.8%, 353/739) compared to females (43.4%, 295/680), and this trend was consistently observed across most grade levels, particularly in elementary school—most notably in the 1st grade (56 males vs. 33 females). However, this apparent gender difference was not statistically significant [*χ*^2^(1) = 2.744, *p* = 0.098], and the negligible effect size (Phi = 0.044) further indicated a weak association between gender and foot abnormality status.

Chi-square tests indicate a statistically significant difference in the distribution of foot abnormality prevalence across different grades [*χ*^2^(11) = 36.98, *p* < 0.001]. Standardized residual analysis, combined with descriptive prevalence data, delineates a distinct developmental pattern for foot abnormalities across school grades. The prevalence peaks strikingly in first grade at 62.7% (standardized residual = + 3.0, *p* < 0.05), indicating a significantly higher-than-expected case load at school entry. Following this peak, a consistent and rapid declining trend is observed through elementary school, from 50.8% in second grade to 39.8% in sixth grade, despite a non-significant minor rebound in fifth grade (53.0%, standardized residual = + 1.5, *p* > 0.05). During junior high (grades 7–9) and the early years of senior high (grades 10–11), prevalence stabilizes at a comparatively lower plateau, ranging between 33% and 43%, with no significant deviations from expected values. An apparent rebound to 61.1% is noted in twelfth grade; however, this finding is based on a very small sample (*N* = 18) and lacks statistical significance (standardized residual = + 1.0), necessitating cautious interpretation due to potential sampling error (Table 1).

**Figure 3:**
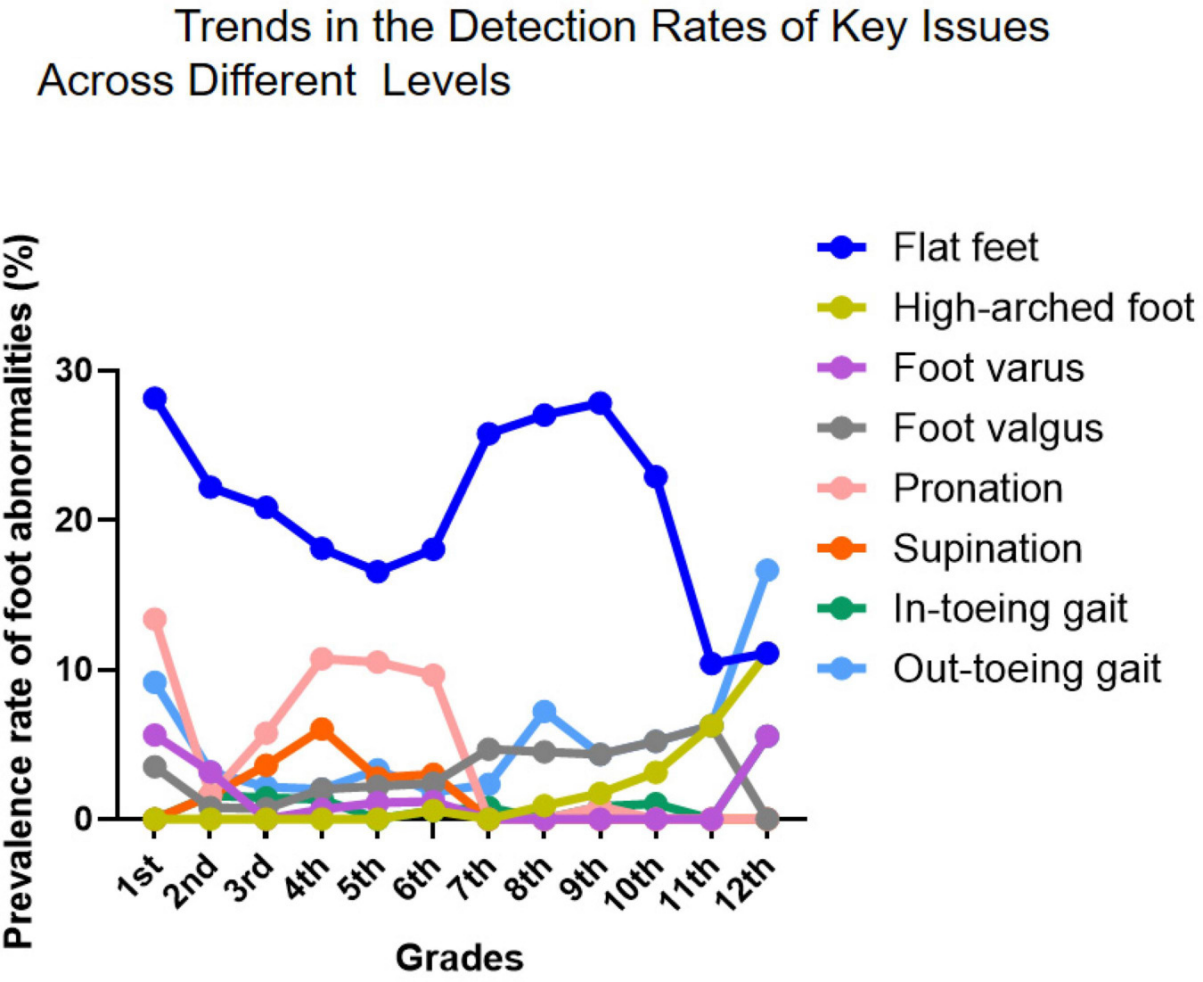
Trends in the Detection Rates of Key Issues Accross Different Levels.

The longitudinal analysis of foot abnormalities across grade levels revealed distinct developmental trajectories for various podiatric conditions. Health assessment abnormalities (abnormal appearance and physiological pain) primarily occurred in lower grade levels, with male students showing higher incidence rates than their female counterparts (Supplementary Table S5). Flat Feet were the most common podiatric disorder, with comparable prevalence between genders. The prevalence peaked in the first grade of elementary school, declined thereafter, showed a secondary peak during junior high school, and decreased again in later high school years, indicating stage-specific characteristics in arch development (Supplementary Table S5 and Figure 3). Conversely, high-arched foot demonstrated an inverse developmental pattern, absent in early grades but reaching 11.11% prevalence by 12th grade (Supplementary Table S5).

Gait Abnormalities showed marked gender differences: Out-toeing gait prevalence was significantly higher in males across all grades, while in-toeing gait was generally rare but showed a notable increase among females in the 12th grade (Supplementary Table S5 and Figure 3). Among Alignment Disorders, pronation was predominantly female, while varus, valgus, and supination were slightly more prevalent in males. Foot valgus was persistently detected across all grades with an atypical surge during high school (Supplementary Table S5). Regarding Structural Deformities, Hallux Valgus peaked in grades 5–7, with incidence significantly higher in females. Calcaneal Valgus was concentrated in elementary school years and showed absolute female predominance (Supplementary Table S5).

**Figure 4:**
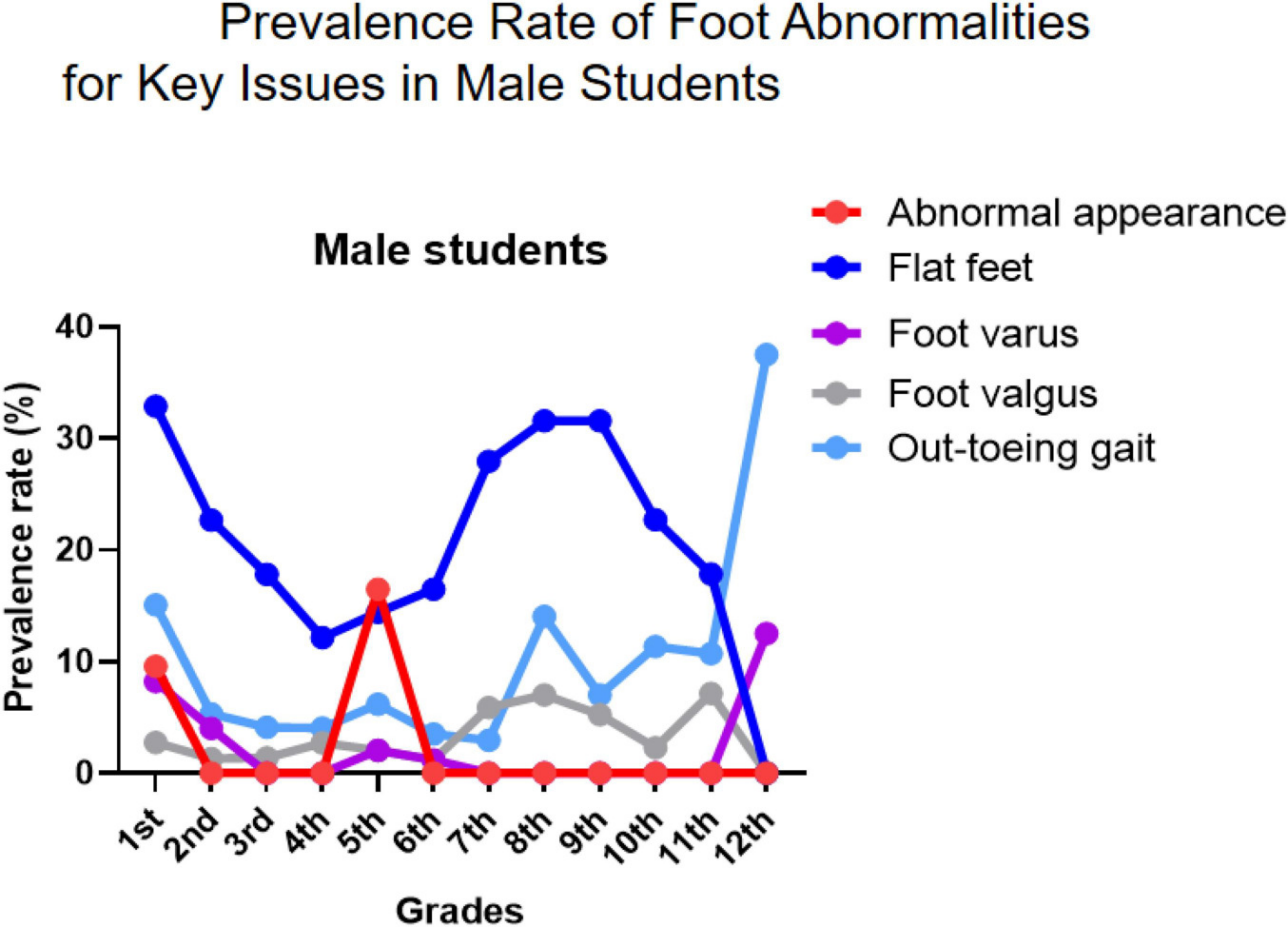
Prevalence rate of foot abnormalities for Key issues in male students.

Intervention priorities for males should focus on flat feet (persistently high across multiple stages), out-toeing gait (significantly elevated at key transitional grades), and the prevalent foot valgus. Recommended intervention windows are early elementary years (flat foot management), fifth grade (appearance abnormality intervention), and key transitional grades (gait correction) (Figure 4).

**Figure 5:**
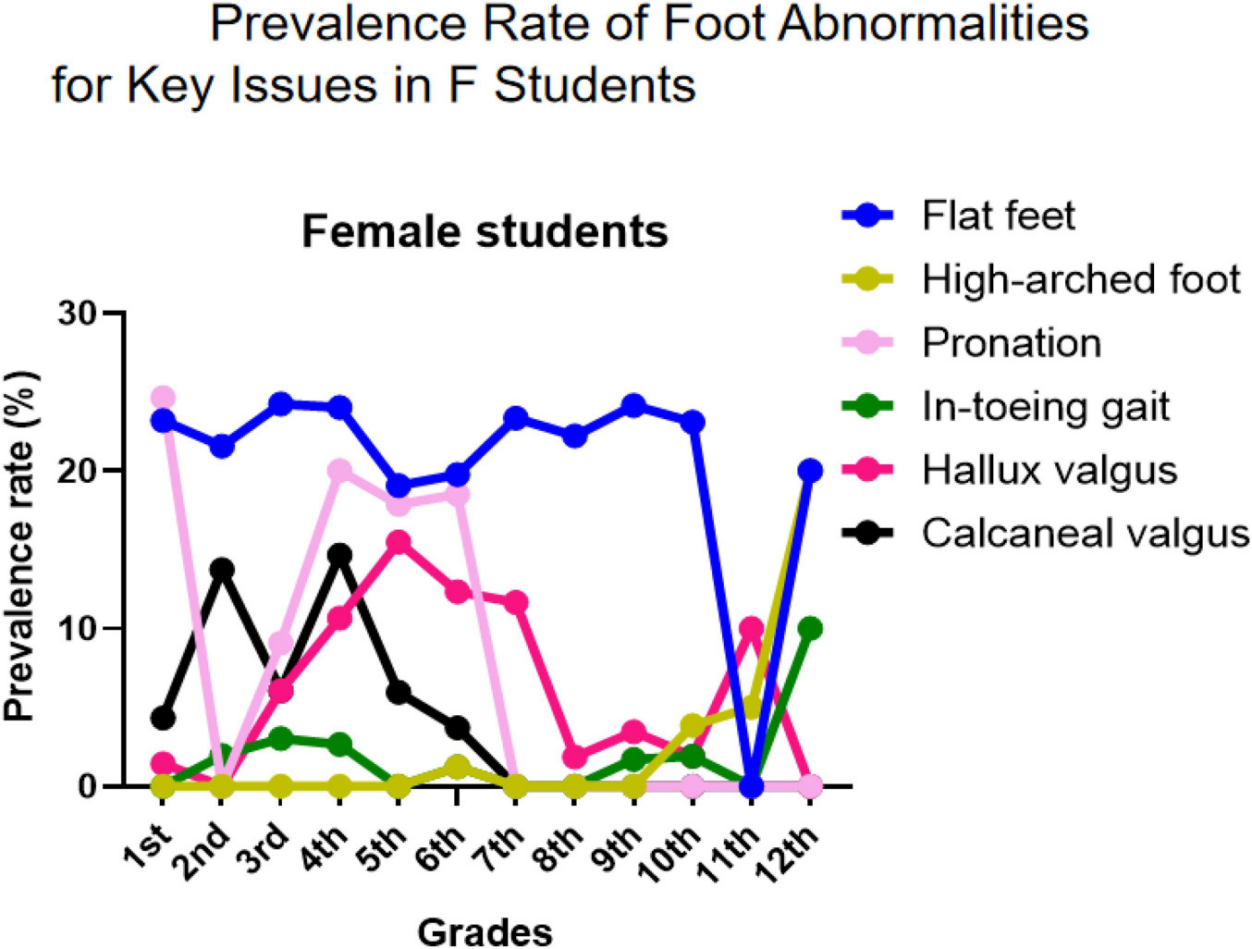
Prevalence rate of foot abnormalities for Key issues in F students.

Intervention priorities for females require particular attention to flat feet (consistently high across grades), pronation (predominant in elementary school), hallux valgus (high in grades 4–7), and calcaneal valgus (concentrated in elementary years). Additionally, high-arched feet and in-toeing gait warrant attention during high school (Figure 5).

**Figure 6:**
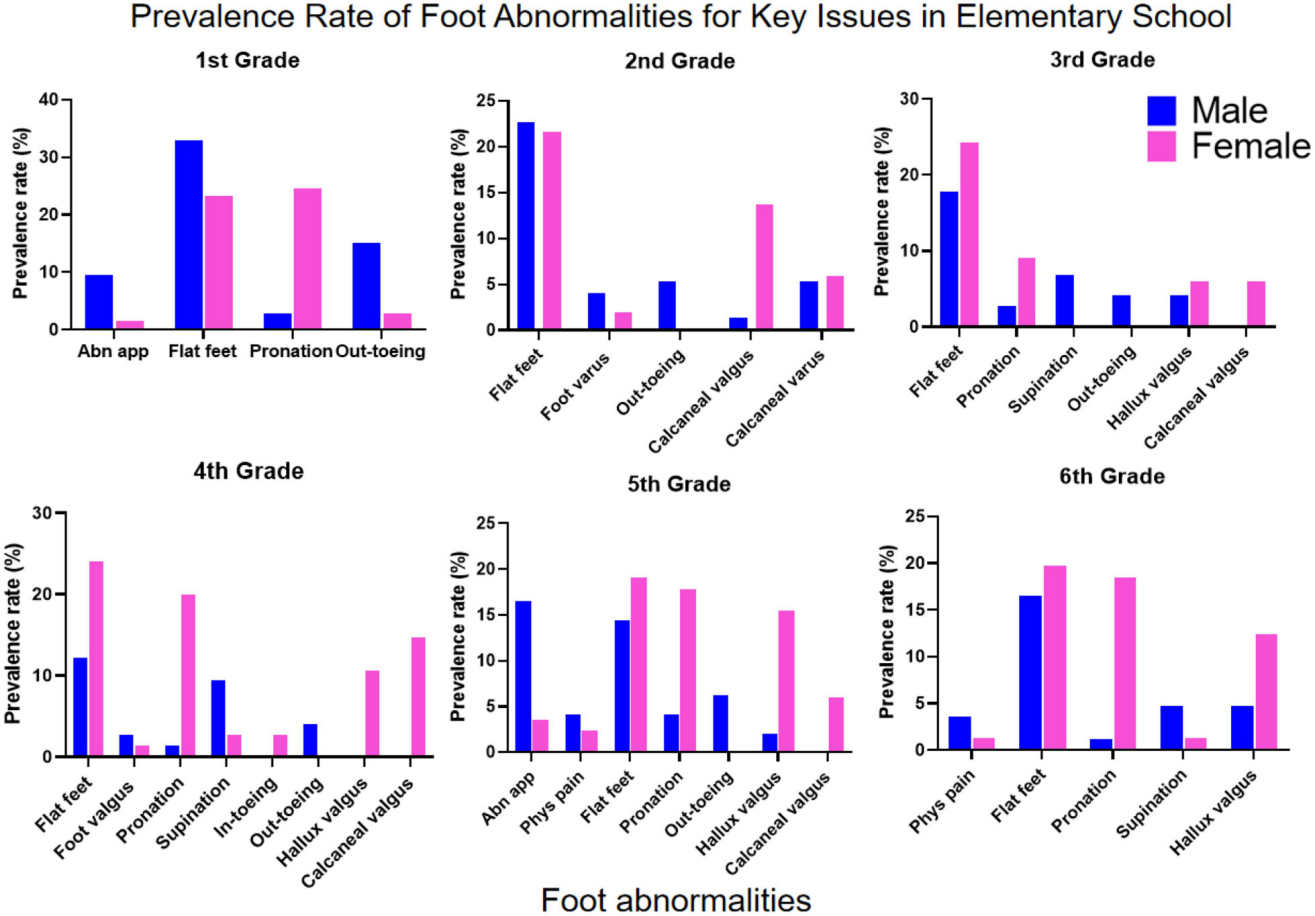
Prevalence rate of foot abnormalities for Key issues in elementary school.

**Figure 7:**
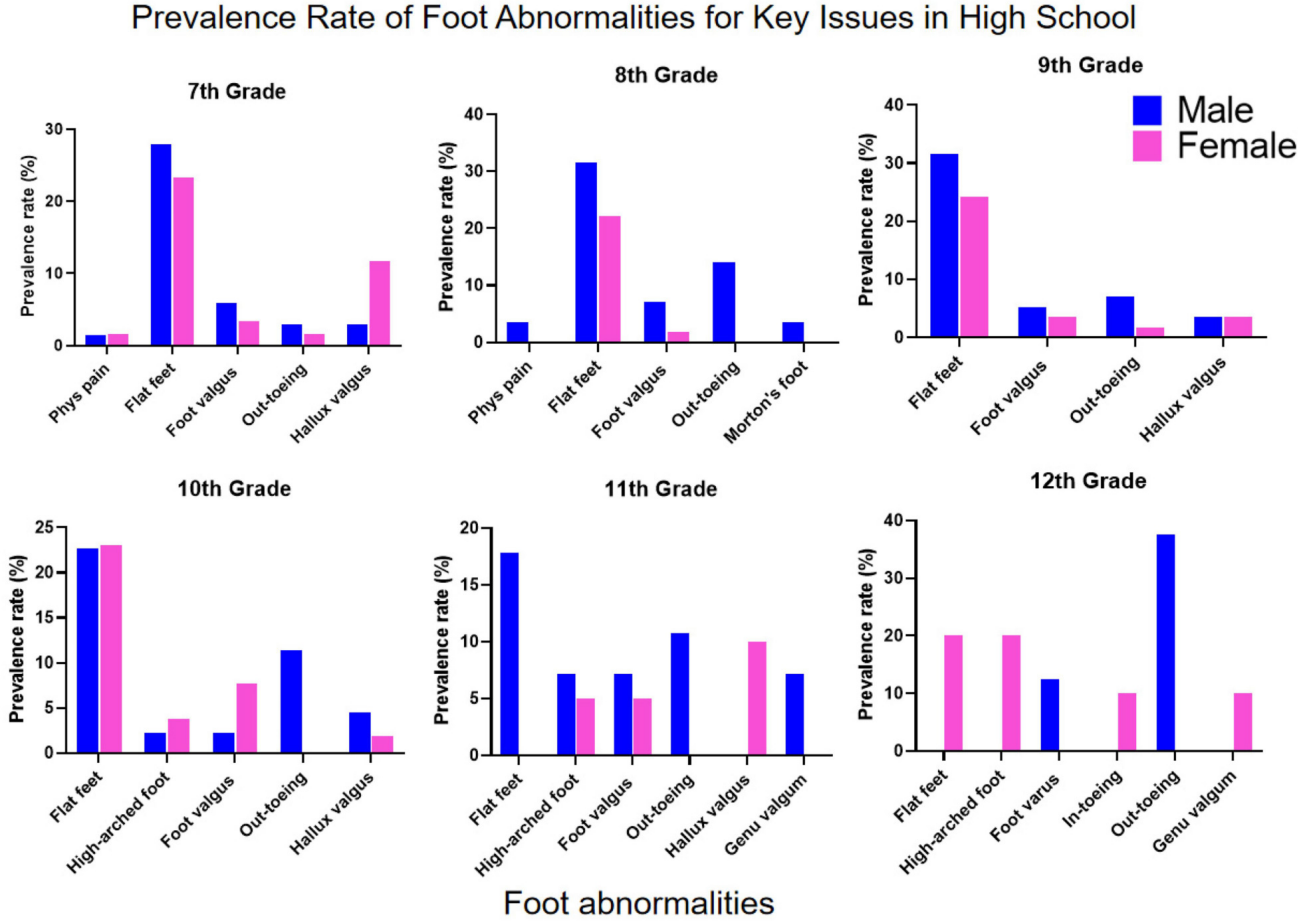
Prevalence rate of foot abnormalities for Key issues in high school.

For elementary school screening, it is recommended that as the variety of foot abnormalities increases with grade level, lower grades should focus on screening for appearance abnormalities, flat feet, and pronation or out-toeing gait, while middle and upper grades should additionally screen for hallux valgus, calcaneal abnormalities, and physiological pain, with clear gender differences necessitating targeted screening (Figure 6 and Table 3). For middle and high school screening, gender-specific patterns persist, requiring ongoing monitoring of flat feet, foot valgus, and out-toeing gait for males, and intensified surveillance for hallux valgus in addition to flat feet for females, while high school students should also be monitored for high-arched feet and in-toeing gait (Figure 7, Table 3).

**Table 3:** Prevalence and gender distribution of foot abnormalities among adolescents in Beijing .

Category	Foot abnormalities	Total	M	F	1st		2nd		3rd		4th		5th		6th		7th		8th		9th		10th		11th		12th	
					M	F	M	F	M	F	M	F	M	F	M	F	M	F	M	F	M	F	M	F	M	F	M	F
Health Assessment	Abnormal appearance	27	23	4	9.6	1.5	0	0	0	0	0	0	16.5	3.6	0	0	0	0	0	0	0	0	0	0	0	0	0	0
	Physiological pain	20	14	6	1.4	1.5	2.7	0	1.4	1.5	0	0	4.1	2.4	3.5	1.2	1.5	1.7	3.5	0	0	0	0	0	0	0	0	0
Foot Arch Abnormalities	Flat feet	308	161	147	32.9	23.2	22.7	21.6	17.8	24.2	12.2	24.0	14.4	19.1	16.5	19.8	27.9	23.3	31.6	22.2	31.6	24.1	22.7	23.1	17.9	0	0	20
	High-arched foot	12	6	6	0	0	0	0	0	0	0	0	0	0	0	1.2	0	0	1.8	0	3.5	0	2.3	3.9	7.1	5.0	0	20
Foot Alignment Disorders	Foot varus	18	13	5	8.2	2.9	4.0	2.0	0	0	0	1.3	2.1	0	1.2	1.2	0	0	0	0	0	0	0	0	0	0	12.5	0
	Foot valgus	42	23	19	2.7	4.4	1.3	0	1.4	0	2.7	1.3	2.1	2.4	1.2	3.7	5.9	3.3	7.0	1.9	5.3	3.5	2.3	7.7	7.1	5.0	0	0
	Pronation	81	13	68	2.7	24.6	2.7	0	2.7	9.1	1.4	20	4.1	17.9	1.2	18.5	0	0	0	0	1.8	0	0	0	0	0	0	0
	Supination	26	21	5	0	0	2.7	0	6.9	0	9.5	2.7	3.1	2.4	4.7	1.2	0	0	0	0	0	0	0	0	0	0	0	0
Gait & Postural Abnormalities	In-toeing gait (Pigeon toe)	11	2	9	0	0	1.3	2.0	0	3.0	0	2.7	0	0	0	1.2	1.5	0	0	0	0	1.7	0	1.9	0	0	0	10
	Out-toeing gait (External rotation)	59	55	4	15.1	2.9	5.3	0	4.1	0	4.1	0	6.2	0	3.5	0	2.9	1.7	14.0	0	7.0	1.7	11.4	0	10.7	0	37.5	0
Local Structural Deformities	Hallux valgus (Bunion deformity)	66	17	49	0	1.5	2.7	0	4.1	6.1	0	10.7	2.1	15.5	4.7	12.4	2.9	11.7	0	1.9	3.5	3.5	4.6	1.9	0	10	0	0
	Calcaneal valgus (Everted heel)	34	1	33	0	4.4	1.3	13.7	0	6.1	0	14.7	0	6.0	0	3.7	0	0	0	0	0	0	0	0	0	0	0	0
	Calcaneal varus (Inverted heel)	8	4	4	0	0	5.3	5.9	0	0	0	1.3	0	0	0	0	0	0	0	0	0	0	0	0	0	0	0	0
	Genu valgum (Knock knees)	6	3	3	0	0	0	0	0	0	0	0	0	0	0	0	0	0	0	1.9	1.8	0	0	1.9	7.1	0	0	10
Special Conditions	Morton's foot (Long second metatarsal)	5	3	2	1.4	0	0	2.0	0	0	0	0	0	0	0	0	0	1.7	3.5	0	0	0	0	0	0	0	0	0

Frequency table demonstrating the distribution of foot abnormalities stratified by: Disorder category: Structural deformities (flat feet, high arches), Alignment disorders (varus/valgus, pronation/supination), Gait abnormalities (in-toeing/out-toeing), Special conditions (Morton's foot). Demographics: Total cases (Total), Gender distribution (M = Male, F = Female), Prevalence rates (%) by subgroup.

## 4 Discussions

### 4.1 Scoliosis screening

AIS is a spinal deformity most frequently observed in females and often progresses most rapidly during periods of rapid skeletal growth. The spine serves as a central mechanical axis, connecting the cranium superiorly and the pelvis inferiorly. This spinal-pelvic complex not only transmits body weight to the lower limbs but also forms the biomechanical foundation for lower-extremity movement (65). This large-scale screening study of 1,419 adolescents in Beijing provides critical insights into the prevalence and epidemiological characteristics of AIS within a representative Chinese urban population. The overall prevalence of 2.04% (Table 1) aligns with previously reported rates in some East Asian cohorts, though it falls at the lower end of the spectrum, which may reflect regional variations or methodological differences in screening protocols (5, 7). The most salient finding of this investigation is the profound and progressively increasing gender disparity, with females exhibiting a prevalence five times greater than that of males (3.53% vs. 0.68%, *p* < 0.001). This discrepancy not only corroborates the well-established female predisposition to AIS but also demonstrates how this divergence magnifies dramatically during secondary education, with female prevalence exceeding 5% in grade 9–12 (Figure 1). This escalating pattern is consistent with the hypothesis that hormonal and biomechanical changes during female puberty may act as significant contributors to curve progression.

Furthermore, our data reveal distinct developmental patterns, identifying specific critical periods of vulnerability. The notably low prevalence in early elementary grades (0.67%–0.79% in grades 1–4) is followed by a sharp increase beginning in grades 5–6 (Table 1). The observed peaks in prevalence in 6th, 9th, and 12th grades (Figure 1) are associated with phases of rapid skeletal growth, a period during which the emergence or progression of spinal deformities may be more likely. This pattern coincides with concurrent lifestyle transitions characteristic of these educational stages, including increases in sedentary behavior and academic demands, factors that could plausibly influence core muscle development and postural control (63, 64). The bimodal distribution of more severe curvatures (≥4°), with clusters in upper elementary (grades 4–6) and middle school (grade 9) grades (Table 2), further underscores these periods as targets for preventive screening.

Beyond the factors discussed above, our findings—particularly the peak prevalence rates at specific grade levels and the marked gender disparity—are consistent with the multifactorial etiological model of AIS. This model posits that in genetically predisposed individuals (e.g., females carrying certain genetic variants such as those in the WFS1 gene, which is involved in mitochondrial function and growth regulation), the rapid growth spurt during puberty may serve as a “second hit” that triggers the development of clinically significant spinal curvature (66). Although the present study did not include genetic testing, future screening models could incorporate genetic risk assessment to better identify high-risk individuals.

The scoliometer measurements offer a nuanced view of spinal health in this population. While the vast majority of adolescents (92.46%) presented with spinal asymmetries of ≤3°, the gender-specific distribution of higher-angle curvatures is striking (Supplementary Table S2). Females accounted for 73.83% of all curvatures ≥4° and all identified 6° cases (Supplementary Table S3), solidifying the link between female sex and curve severity. The subgroup analyses pinpoint specific at-risk groups, such as 5th-grade males for 4° curves and 9th-grade females for 5° curves (Supplementary Table S4), providing actionable data for refining school-based screening programs to focus on high-risk demographics.

In conclusion, our findings confirm that the risk for AIS is not static but evolves throughout adolescence, heavily influenced by gender and pubertal development. These results advocate for the implementation of a targeted, two-tiered screening strategy: initial screenings should commence prior to the onset of the growth spurt, with particularly vigilant follow-up and monitoring for females throughout secondary school. This approach promises to optimize resource allocation and improve early detection rates, ultimately mitigating the long-term impact of scoliosis through timely intervention.

### 4.2 Foot and gait implications

Ankle and foot development is a dynamic process. During childhood, the arch gradually forms, and by adolescence, its function matures (67). Most structural variations before the age of 18 are part of normal development and typically require no intervention. However, medical attention is needed if symptoms such as pain, limited mobility, or scoliosis are present. Foot and ankle disorders can lead to various functional impairments. Flat feet or high arches may cause knock knees (valgus) or bow legs (varus), increasing the risk of knee injuries. Tight Achilles tendons often contribute to shin or heel pain. Arch collapse can reduce running efficiency, while weakness may impair the ability to stop or change direction quickly during sports. Furthermore, these issues can lead to compensatory postural changes and even contribute to chronic low back pain.

The detection rate of flatfoot in children and adolescents shows significant gender and age differences, with a higher prevalence among males (52%) than females (36%) (44), likely due to earlier arch development in females and thicker plantar fat pads in males. The incidence of flatfoot decreases markedly with age, particularly between ages 3–6 and 7–12. Martin Pfeiffer (44) reported flatfoot in 54% of 3-year-olds compared to only 24% of 6-year-olds. A 2020 cross-sectional survey by Yohanes (46) also found that younger children had a higher probability of flatfoot. Other studies indicate a decline in incidence from 72.6% to 37.9% between ages 7 and 12 (47), reflecting the natural maturation of the arch. Environmental and lifestyle factors also play an important role. Urban children show a significantly higher prevalence of flatfoot than rural children—51.2% vs. 35% (48)—which is associated with greater use of closed-toe shoes, less physical activity, and higher rates of overweight and obesity; obesity can triple the risk of flatfoot compared to normal-weight children (44). In our study, we revealed a high overall rate of foot abnormalities (45.67%, Table 1), far exceeding that of spinal deformities in the same group. A notably high rate of foot abnormalities was observed in early childhood (Figure 3), especially among first graders (62.68%, Table 1), with a general decline throughout elementary school. This suggests that many early foot conditions may resolve naturally with musculoskeletal strengthening and gait refinement. However, a secondary rise in junior high and atypical peaks in senior high (e.g., 61.11% in grade 12, Table 1) indicate a non-linear developmental trajectory, likely influenced by pubertal growth spurts, sedentary behaviors, academic pressures, and changes in physical activity.

**Figure 8:**
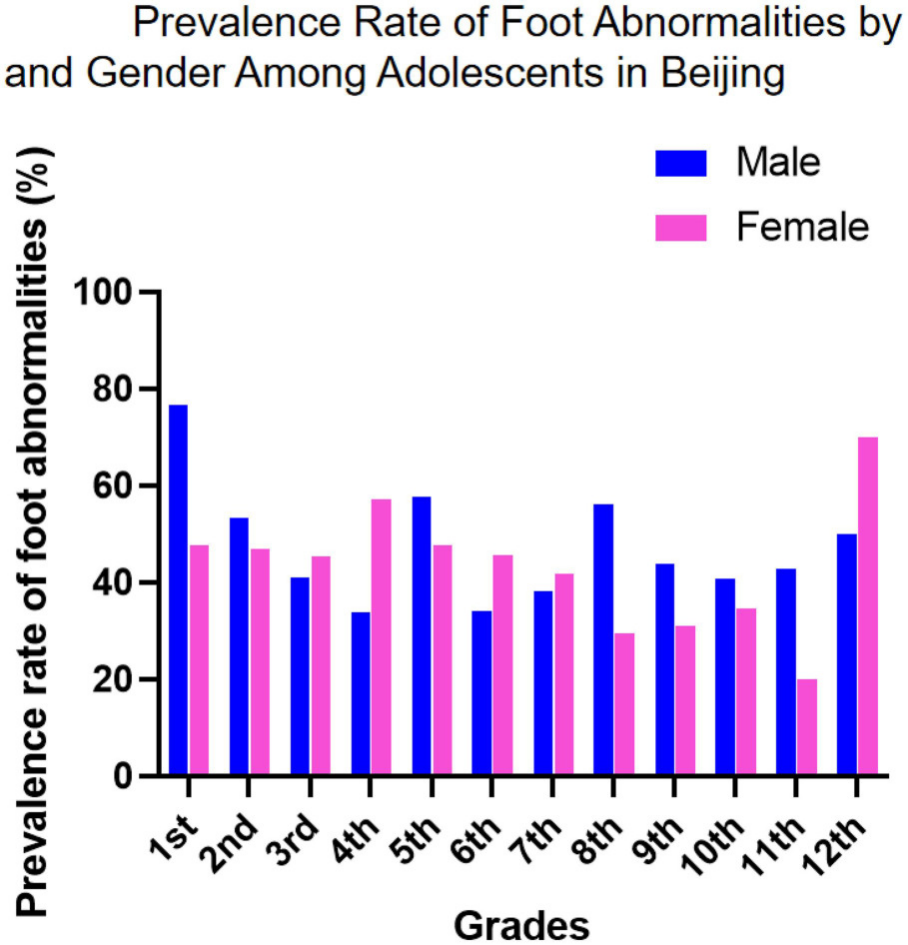
Prevalence rate of foot abnormalities by and gender Among adolescents in Beijing.

A consistent gender disparity was observed, with males showing higher overall prevalence (Figure 8), particularly in elementary school (Figures 4, 6, 7, 8). This may stem from biomechanical differences, variations in musculoskeletal maturation, and activity patterns. Exceptions such as higher rates of hallux valgus, pronation, and calcaneal valgus in females (Figures 5–7) point to possible sex-specific vulnerabilities related to ligament laxity, footwear, or other biological factors. Flatfoot was the most common disorder, affecting over one-fifth of participants, with similar rates across genders. Its biphasic pattern—an initial decline after first grade followed by a rise in junior high—suggests two etiological phases: early architectural immaturity and later influences such as weight gain, rapid growth, or altered biomechanics in adolescence. In contrast, high-arched feet increased progressively with age, indicating divergent developmental pathways. Analysis of dynamic gait abnormalities revealed pronounced gender differences. Out-toeing was more common in males, especially during transitional grades, suggesting differences in femoral or tibial torsion or neuromuscular control. In-toeing, though less frequent, was more often seen in females (Table 1).

These findings support the need for school-based screening that prioritizes flatfoot detection in early elementary and junior high years. Gender-specific protocols should focus on alignment and gait in males and hallux valgus in females, with continued podiatric surveillance into adolescence to address late emerging conditions such as high arches and gait changes. Healthy arch development is essential for children's growth. To support normal arch formation and prevent flatfoot, children should be encouraged to engage in appropriate physical activities (e.g., jumping) and spend more time barefoot. Prolonged sitting and excessive weight-bearing should be avoided. Parents should choose well-fitted shoes and help maintain a healthy weight, especially during critical growth periods. Activities with high impact on the arches should be avoided to prevent injury. Arch supports may be used for symptomatic flatfoot to improve pressure distribution (68, 69).

### 4.3 Prevention and intervention recommendations

To effectively mitigate AIS, a comprehensive strategy is recommended, centered on a two-tiered school-based screening program initiated before the adolescent growth spurt (around grade 5), with intensified focus on female students—who show a fivefold higher prevalence—particularly during high-risk grades (6, 9, and 12) associated with rapid skeletal development. This should be supplemented by systematic follow-up protocols prioritizing females in secondary education for close monitoring during peak pubertal growth to enable early detection of curve progression. Concurrently, postural health education should be integrated into school curricula, especially during key educational transitions, to reduce sedentary behavior risks and promote core-strengthening activities that enhance spinal stability.

For foot health, systematic foot screenings are recommended during key developmental windows, such as lower elementary and junior high school stages, to detect early flatfoot and adolescent-onset secondary abnormalities. Age-appropriate physical activities—such as jumping and barefoot activities on safe surfaces—should be encouraged to strengthen the arch, while sedentary behavior and high-impact exercises should be avoided. Parents should be guided to choose well-fitted supportive footwear and to manage their children's weight, with particular emphasis on addressing overweight and sedentary lifestyles among urban children. For symptomatic cases, arch supports can be used to improve biomechanical load distribution, and early physical therapy or rehabilitative exercise should be provided to those with gait abnormalities such as in-toeing or out-toeing.

### 4.4 Study limitations and future directions

Despite the valuable insights provided by this large-scale screening study, several limitations should be considered. First, the cross-sectional design precludes the establishment of causal relationships between observed factors—such as lifestyle changes, footwear, or physical activity—and the development of spinal or foot disorders. Second, the sample sizes in senior high school grades (e.g., 11th and 12th grades) were relatively small, which may affect the precision and generalizability of prevalence estimates in these age groups. Additionally, although standardized clinical tools were used (e.g., scoliometer, FPI-6, plantar pressure analysis), the study did not incorporate radiographic confirmation, which remains the gold standard for diagnosing structural deformities such as AIS and certain foot pathologies. Furthermore, potential confounding variables, including detailed physical activity history, genetic predisposition, and nutritional status, were not comprehensively assessed.

Future research should prioritize longitudinal studies to track the natural progression of both spinal and foot conditions from childhood through adolescence, enabling the identification of critical windows for intervention. There is also a need to integrate advanced imaging modalities with biomechanical assessments to better understand the structural and functional aspects of these disorders. Investigating the interplay between environmental factors—such as schoolbag weight, daily activity patterns, and footwear type—and biomechanical development could inform more effective public health strategies. Moreover, prospective studies evaluating the efficacy of targeted interventions, such as muscle strengthening programs, footwear modifications, and early orthotic use, are essential to translate screening findings into actionable clinical protocols. Finally, expanding screening efforts to diverse geographical and socioeconomic settings will help validate the generalizability of the current findings and support the development of tailored prevention strategies across different populations.

In addition, a promising avenue for future etiological research lies in the integration of multi-omics data and causal inference methodologies. Combining genomics, radiomics, and deep clinical phenotyping could uncover novel biomarkers and pathogenic pathways underlying AIS and foot biomechanical disorders. In particular, Mendelian randomization (MR) offers a powerful framework to move beyond the correlational nature of cross-sectional studies. By using genetic variants as instrumental variables, MR can help infer potential causal relationships between modifiable risk factors (such as vitamin D levels, physical activity, or sedentary time) and the development of AIS (70, 71). This approach can provide more robust evidence to guide primary prevention strategies. Future studies employing MR designs are encouraged to clarify these causal links and identify actionable targets for intervention.

## 5 Conclusion

This study underscores the dynamic and multifactorial nature of musculoskeletal development during adolescence, with distinct epidemiological and biomechanical patterns observed for both adolescent AIS and foot disorders. We identified a significant gender disparity in AIS prevalence, with females exhibiting a fivefold higher rate than males, particularly during pubertal growth phases. Critical developmental windows—specifically grades 5–6 and secondary education periods—were associated with increased risk, likely due to interactions between rapid skeletal growth, hormonal changes, and lifestyle factors such as sedentary behavior. Similarly, foot abnormalities demonstrated high prevalence (45.67%), with clear gender- and grade-specific trends: males showed higher susceptibility to flatfoot, especially in early grades, whereas females exhibited greater predisposition to certain structural and alignment issues such as hallux valgus in later school years. The decline in foot abnormalities with age suggests partial natural resolution through maturation, though secondary surges during adolescence highlight the ongoing influence of growth and environmental factors. These findings advocate for a dual-focused public health strategy: implementing targeted, stage-specific screening protocols for spinal and foot health—particularly during key pubertal transitions—and promoting preventive measures including age-appropriate physical activity, rational footwear use, and weight management. Such an approach may significantly improve early detection, inform timely interventions, and reduce the long-term burden of musculoskeletal disorders in the adolescent population.

## Data Availability

The original contributions presented in the study are included in the article/Supplementary Material, further inquiries can be directed to the corresponding author.
